# Potential roles of enteric glial cells in Crohn's disease: A critical review

**DOI:** 10.1111/cpr.13536

**Published:** 2023-08-08

**Authors:** Xinyi Mao, Jun Shen

**Affiliations:** ^1^ Division of Gastroenterology and Hepatology Baoshan Branch, Renji Hospital, School of Medicine, Shanghai Jiao Tong University Shanghai China; ^2^ Division of Gastroenterology and Hepatology, Key Laboratory of Gastroenterology and Hepatology Ministry of Health, Inflammatory Bowel Disease Research Center, Renji Hospital, School of Medicine, Shanghai Jiao Tong University, Shanghai Institute of Digestive Disease Shanghai China

## Abstract

Enteric glial cells in the enteric nervous system are critical for the regulation of gastrointestinal homeostasis. Increasing evidence suggests two‐way communication between enteric glial cells and both enteric neurons and immune cells. These interactions may be important in the pathogenesis of Crohn's disease (CD), a chronic relapsing disease characterized by a dysregulated immune response. Structural abnormalities in glial cells have been identified in CD. Furthermore, classical inflammatory pathways associated with CD (e.g., the nuclear factor kappa‐B pathway) function in enteric glial cells. However, the specific mechanisms by which enteric glial cells contribute to CD have not been summarized in detail. In this review, we describe the possible roles of enteric glial cells in the pathogenesis of CD, including the roles of glia–immune interactions, neuronal modulation, neural plasticity, and barrier integrity. Additionally, the implications for the development of therapeutic strategies for CD based on enteric glial cell‐mediated pathogenic processes are discussed.

## Graphical abstract

Crohn's disease (CD) is determined by complicated interactions among genetic and environmental and the gut microbiota, resulting in dysregulated innate and adaptive immune responses. There are multiple roles of enteric glial cells (EGCs) in the pathogenesis of CD, including the roles of glia–immune interactions, neuronal modulation, neural plasticity, and barrier integrity. Potential therapeutic strategies for CD based on enteric glial cells‐mediated pathogenic processes can be used in the future.

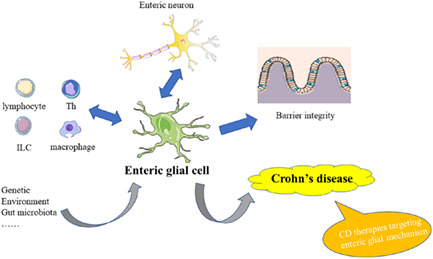

## 1 INTRODUCTION

Inflammatory bowel disease (IBD), encompassing Crohn's disease (CD) and ulcerative colitis (UC), is a chronic relapsing disease mainly affecting the gastrointestinal tract.^1^ CD is determined by complicated interactions among genetics and environmental and gut microbiota, resulting in dysregulated innate and adaptive immune responses.^1^ Although the specific pathogenesis of CD is still unclear, increasing studies have focused on the imbalance between pro‐inflammatory and anti‐inflammatory molecules, and the potential roles of cytokines in CD treatment.^2^ Anti‐inflammatory mediators, which are derived from omega 3 fatty acids, eicosapentaenoic (EPA) and docosahexaenoic (DHA) acids further produce E‐series resolvins, D‐series resolvins (RvD), D‐protectins, and macrophage mediator in pro‐resolving inflammation (maresins).^3^ They collectively activate several cellular and molecular processes that spontaneously attenuate inflammation and restore tissue homeostasis, which may impact IBD.^3^, ^4^ Resolvins may also have a therapeutic potential for decreasing visceral pain, a typical symptom of CD.^3^

Enteric glial cells, a component of the enteric nervous system (ENS), are comparable in morphology and function to astrocytes in the central nervous system (CNS).^5^ These are miniscule, star‐shaped cells that enclose enteric neuronal cell bodies and multi‐axonal bundles.^6^ They extend their processes at the ganglionic edge into the intestinal mucosa and firmly anchor themselves.^7^ Furthermore, enteric glial cells build a dense structure of cells that surround and mechanically support enteric neurons in the ENS,^8^ similar to astrocytes, which also support neuron functions in the CNS.^9^ Moreover, enteric glial cells can modulate gut motility, maintain barrier integrity, control the immunological response, and regulate intestinal inflammation.^10^

Enteric glial cells contribute to metabolism and provide structure for enteric neurons.^11^, ^12^ Notably, there is growing evidence for bidirectional interactions between enteric glial cells and neurons in the regulation of essential gastrointestinal functions.^13^, ^14^, ^15^ The dysregulation of this interaction disrupts intestinal homeostasis and may be involved in the pathogenesis of various gastrointestinal diseases, such as IBD.^16^ Enteric glia interact with the intestinal epithelium, immune cells, endothelial cells, and enteric neurons, all of which are likely involved in the pathophysiology of CD.^6^ Furthermore, glial cell structural abnormalities in the ENS have been identified in patients with CD.^17^ Moreover, in patients with CD, both noninvolved and involved ileal and colonic samples exhibited a disrupted enteric glial cell network.^18^ In a double transgenic mouse model, Cornet et al.^18^ identified the cause of intestinal inflammation as a CD8^+^ T cell‐mediated autoimmune response that targets enteric glial cells. Classical inflammatory pathways in CD also involve intestinal glial cells, including the nuclear factor kappa‐B (NF‐κB) pathway, which promotes the expression of pro‐inflammatory cytokines, such as tumour necrosis factor‐alpha (TNF‐α), interleukin (IL)‐1, and IL‐6^19^ and contributes to inflammation‐related tissue damage.^20^ In patients with CD, NF‐κB promotes an increase in polymorphonuclear cells in the lamina propria, neutrophils in the epithelium and prominent granuloma formation.^21^

This review summarizes the possible roles of enteric glial cells in CD, associated signalling pathways with neuromodulatory, immunomodulatory, and barrier‐protective roles, as well as promising therapies targeting enteric glial cells.

## 2 ENTERIC GLIAL CELL–IMMUNE INTERACTIONS IN CD

**FIGURE 1:**
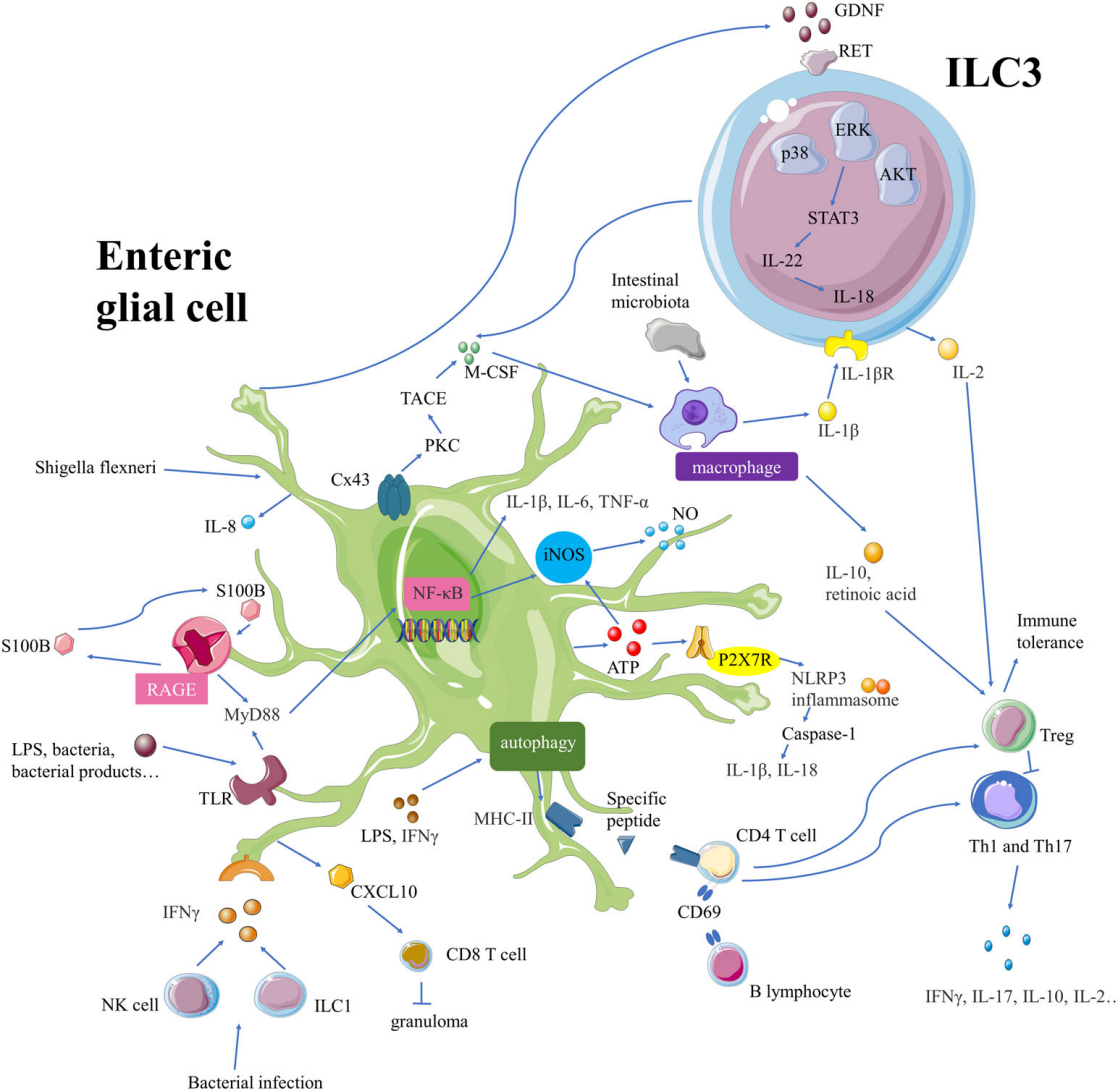
Overview of the role of enteric glial cells in intestinal immune homeostasis. First, LPS, bacteria, and bacterial products activate TLR‐S100B‐RAGE signalling in enteric glial cells, subsequently activating the MyD88 and NF‐κB pathways. Following this, iNOS is induced, and NO is released. ATP can activate P2X7R and the NLRP3‐inflammasome. *Shigella flexneri* bacterial infection can also activate enteric glia. Second, GDNF activates ILC3 and regulates the p38‐ERK‐AKT cascade to activate IL‐22 and IL‐18. Enteric glial cells produce M‐CSF, which interacts with ILC3 and releases regulatory factors. In addition, enteric glial cells express MHC‐II, allowing them to present specific peptides to B lymphocytes and CD4+ T cells. Finally, IFNγ released by NK cells and ILC1 can activate enteric glial cells to release CXCL10 and recruit CD8+ T cells.

Although enteric glial cells are not immune cells, they respond to pathological stimuli, cytokines, and chemokines. Enteric glial cells may play pro‐inflammatory roles in the development of CD. Enteric glial cells can interact with lymphocytes,^22^ macrophages,^23^ natural killer (NK) cells,^24^ and other immune cells to influence gut immune homeostasis. The signalling pathways by which enteric glial cells regulate immune homeostasis are illustrated in Figure 1.

Researchers have identified that adherent‐invasive *Escherichia coli* (AIEC) may have a role in the pathophysiology of IBD^25^; specifically, the interaction between AIEC and enteric glia is a possible influencing factor. AIEC is found in the intestines and mesenteric lymph nodes of 57% of patients with CD.^26^ Moreover, in mucosal biopsies from patients with CD, multiple CD‐related deficiencies in the innate immune system facilitated AIEC‐induced inflammation.^27^ *E. coli* induces inflammatory reactions in host cells by engaging Toll‐like receptors (TLRs). Furthermore, enteric glial cells distinguish between pathogens and probiotics via TLRs.^28^ Enteroglial‐specific S100B increases intestinal inflammation when enteric glial cells are activated.^29^ Bacterial products (e.g., lipopolysaccharides) or pathogenic microorganisms cause S100B release from enteric glial cells.^28^ S100B interacts with TLR, amplifies colonic inflammation, and promotes macrophage recruitment in the mucosa.^29^ Receptor for advanced glycation end products (RAGE), a cell surface protein, binds to proinflammatory cytokine‐like mediators of the S100/calgranulin family.^30^ TLR expression depends on the type of bacteria in contact with enteric glial cells, and nitric oxide (NO) production depends on both TLR and S100B‐RAGE expression.^28^ The S100B/RAGE complex interacts with MyD88, a downstream mediator of the TLR signalling pathway, and activates NF‐κB protein expression. Consequently, this stimulates inducible nitric oxide synthase (iNOS) expression and NO release.^28^ Furthermore, NO production was increased in a mouse model of colitis, and the dysregulation of intestinal ion transport was caused by increased inducible NO synthase (NOS2) synthesis in enteric glia.^31^ NO triggers activated macrophage‐induced cytotoxicity and improves the anti‐bacterial response.^32^ Additionally, normal gut physiology is regulated by adenosine triphosphate (ATP) and NO, both of which can be released by enteric glia (see details in the next section). During inflammation, purine signalling can increase ATP release by enteric glial cells and also promote enhanced iNOS activity and NO release.^33^, ^34^, ^35^

Besides, NLRP3/inflammasome alterations are linked to CD susceptibility.^36^ ATP can act on P2X7 purine receptors (P2X7R) to trigger K+ efflux and the subsequent assembly of the NLPR3‐inflammasome.^37^, ^38^, ^39^ The NLRP3‐inflammasome includes NACHT LRR protein (NLRP) and apoptosis‐associated speck‐like protein containing CARD (ASC), which cleaves procaspase‐1 (pro‐casp‐1) into caspase‐1. The cleavage of pro‐IL‐18 and pro‐IL‐1β into IL‐18 and IL‐1β, respectively, is catalysed by caspase‐1, thereby enhancing chronic intestinal inflammation.^36^, ^40^, ^41^

Bacterial infection by *Shigella flexneri* can also activate enteric glial cells and suggest a protective role of enteric glial cells. Enteroglial‐derived factors such as S‐nitrosoglutathione can protect against encroaching infections.^42^ In an in vitro model, the IL‐8 concentration was decreased when the intestinal epithelial barrier was cocultured with enteric glial cells.^42^

Because the intestine is the largest immune organ, glial cells are well‐positioned to mediate interactions between the immunological and neurological systems.^43^ Essentially, glial cells are the principal regulators of innate immunity and neurological function and can interact with lymphoid cells. Patients with CD have considerably lower counts of group 3 innate lymphoid cells (ILC3s) and T regulatory cells (*T*_reg_s) in the small intestine than corresponding estimates in healthy controls^44^, ^45^, ^46^ and fewer IL‐2^+^ ILC3s in the terminal ileum.^46^ Glial‐derived neurotrophic factors (GDNFs) activate a highly expressed tyrosine kinase receptor, RET, in ILC3.^22^ ILC3s play essential roles in protecting against intracellular bacteria and parasites,^47^ collaborating with other immune cells to limit commensal microorganisms, and regulating host–commensal‐bacteria relationships.^47^

In the inflamed ileum of patients with CD, increased STAT3 activity is related to the pathogenesis of the condition.^48^ Furthermore, IL‐18 is up‐regulated in the peripheral blood and intestinal lesions of patients with CD.^49^ By activating RET in ILC3, the p38‐ERK‐AKT cascade is phosphorylated, which enhances STAT3 phosphorylation. STAT3 phosphorylation consequently induces innate IL‐22 production,^22^ thereby promoting intestinal epithelial reactivity and repair^47^ as well as gut defence and homeostasis.^22^ IL‐22 binds to the IL‐22 receptor to stimulate IL‐18 mRNA overexpression in ileal epithelial cells, which subsequently amplifies Th1‐mediated intestinal inflammation and destroys the mucosal barrier.^50^

In response to pro‐inflammatory stimuli, enteric glia cells produce macrophage colony‐stimulating factor (M‐CSF), inducing persistent visceral hypersensitivity and immune activation, ultimately contributing to abdominal pain in CD.^23^ In a 2,4‐dinitrobenzenesulfonic acid‐induced intestinal damage rat model, enteric glial cell inhibition decreased colitis‐related visceral hyperalgesia by overexpressing S100β and TRPV1 along the pain signal pathway and also prevented intestinal damage.^51^ Glial connexin‐43 (Cx43) hemichannels are required for the induction of protein kinase C (PKC), and subsequently, TNF‐alpha converting enzyme (TACE) is activated to cleave membrane‐bound M‐CSF.^23^ A genome‐wide association study revealed that monocytes preferentially induce CD‐associated genes (e.g., *NOD2*) in response to inflammatory stimuli, and these genes are downregulated during the differentiation of monocytes into macrophages.^52^ Retinoic acid‐related orphan receptor gamma (RORγ) t^+^ ILC3 serves as the main source of granulocyte‐macrophage colony‐stimulating factor (GM‐CSF) in the gut, and ILC3‐derived GM‐CSF production depends on the ability of macrophages to recognize microbial signals and produce interleukin‐1.^53^ ILC3‐derived GM‐CSF induces macrophages to produce retinoic acid (RA) and IL‐10. To maintain immunological tolerance, macrophages convert naive T cells into T_reg_s.^53^ However, more conclusive evidence that the interaction between ILC3 and enteric glial cells is related to CD pathogenesis is still lacking.

Additionally, IBD, including CD, is linked to chronic microbial infection, which generates persistent local inflammation and tissue injury.^54^ MHC‐II regulates intestinal immune responses and is highly expressed in enteric glial cells in patients with CD.^55^ Average IL‐17 cell counts are significantly higher in the mucosa and serum of patients with active CD than in healthy individuals and cases of inactive CD.^56^ Patients with active CD had considerably higher *IL‐10* and *IFNγ* mRNA levels than those of healthy individuals.^57^ In CD, weak immune responses result in chronic tissue damage, intractable infection, and protracted inflammation.^58^ Mice transferred with IBD microbiotas exhibited an increased amount of intestinal T‐helper 17 (Th17) and Th2 cells, with a reduced amount of intestinal RORγt^+^ T_reg_ cells in comparison to microbiotas from healthy donars.^54^ Proinflammatory stimuli, such as IFN‐γ and lipopolysaccharide (LPS), can cause glial cell autophagy.^59^ Enteric glial MHC‐II is derived from products of autophagy, rather than phagocytosed foreign antigens.^59^ Effective CD69^+^ B cell and T cell activation is facilitated by glial MHC‐II expression, with notable impacts on Th17 and T_reg_ subtypes^59^ and the release of proinflammatory factors such as IFN‐γ, IL‐17, IL‐10, and IL‐2. MHC‐II expression by enteric glial cells may help maintain immunological homeostasis in inflammatory and autoimmune diseases.

Recently, Progatzky et al.^24^ have identified a novel signalling pathway that regulates intestinal tissue repair and immunity through enteric glial cells. After *Heligmosomoides polygyrus* infection, NK cells and type 1 innate lymphoid cells (ILC1s) are activated and produce interferon gamma (IFNγ),^24^ which is overexpressed in vitro in human CD explants.^60^ CXCL10 upregulation is an early and rapid enteric glial cell response to IFNγ. CXCL10 is required for enteric glial cell‐mediated repair by recruiting CD8^+^ T cells to boost IFNγ production in the muscularis and inhibit granuloma formation.^24^ Enteric glial cells can also interact with mesothelial cells, fibroblasts, and immune cells.^24^ These lineages play significant roles in the pathogenesis of CD.^61^ The specific mechanisms by which enteric glial cells interact with these cells should have further investigation.

## 3 NEUROPLASTICITY AND NEUROMODULATION OF ENTERIC GLIAL CELLS IN CD

In pathological states, enteric glial cells transform into a “reactive enteric glia” state to react to intestinal inflammation.^62^ The alternations encompass changes to the molecular composition, structure and function and may change glial activities by introducing or removing capabilities that may be advantageous or harmful to nearby neural and non‐neuronal cells.^10^ This response has been observed in various pathophysiological disruptions, including intestinal inflammation and infection.^10^ Enteric glia are a crucial component of inflammation in the gastrointestinal tract and have a significant impact on pathological changes in the ENS. However, the neuroplasticity that is linked to glial cells in gastrointestinal dysfunction and connection between neuroplasticity and inflammation are not entirely understood. Enteric glial cells are crucial in regulating neural‐controlled motor function, motility, and intestinal transit activity.^63^ CD is linked to long‐term gut dysfunction induced by changes in the ENS and a depletion of enteric neurons.^16^

In a 2,4‐dinitrobenzenesulfonic acid model of colitis, enteric glial cells actively contributed to the death of enteric neurons during gut inflammation.^34^ Similarly, Gulbransen et al.^16^ have proposed a mechanism of enteric neuron death during intestinal inflammation using in vivo models of experimental colitis. Purines produced during inflammation cause death of enteric neurons by stimulating P2X7R, resulting in ATP release through neuronal pannexin‐1 (Panx1) channels.^16^ P2X7R controls the release of cytokines and chemokines, the survival and differentiation of T lymphocytes, the activation of transcription factors, and cell death.^64^ Additionally, in vivo models have demonstrated that elevated ATP levels can activate neuronal P2X7R, Panx1, the ASC adaptor protein, and caspases, which can cause inflammation‐induced neuronal cell death.^16^ ATP primarily targets cells expressing hypersensitive P2X7R as an autocrine signal.^65^ The P2X7R/NLRP3 axis has also been linked to pyroptosis, a type of immune cell death initiated by the activation of inflammatory caspases such as caspases 1 and 11 in mice and caspases 1, 4, and 5 in humans.^65^

Purines produced by enteric neurons prior to neuronal death stimulate enteric glia during colitis in vivo.^16^ By stimulating P2Y1Rs in enteric glial cells, eNTPDase2 rapidly hydrolyzes neuronal ATP produced by Panx1 to ADP, which then stimulates intracellular calcium (Ca^2+^) responses in adjacent enteric glial cells.^16^, ^34^ Enteric neuron death can be induced by glial activation alone via the opening of connexin‐43 (Cx43) hemichannels and ATP release.^34^, ^66^ Notably, normal enteric reflexes utilize similar glial recruitment through neurotransmitters and gliotransmitter release processes; however, these mechanisms do not result in neuroinflammation.^67^ It is possible that glial Cx43 hemichannel gating by NO is necessary for glial cell‐driven neuron death.^34^ During inflammation, glial cells directly generate oxidative stressors by activating iNOS,^28^, ^68^ which subsequently generates NO.^69^ NO does not play a significant role in normal neuron–glia communication since iNOS expression in enteric glial cells is only elevated during inflammation.

Although there is still insufficient in vivo evidence, GABA has been implicated in neuroimmune interactions by modulating immune cell activity in various systemic and intestinal inflammatory diseases, like IBD.^70^ Glial Ca^2+^ responses trigger the release of gliotransmitters, including ATP and GABA, through the membrane channels Cx43^34^, ^66^ and GABA transporter 2 (GAT2).^71^, ^72^ GABA is a putative regulator of both excitatory and inhibitory signals in enteric neurons and modulates motor and secretory gastrointestinal activities.^72^

Additionally, diminished enteric glial networks have been observed in the colons of patients with CD,^73^ and plexitis has been linked to early CD recurrence following surgery.^74^ Accordingly, the occurrence of CD may be closely related to the damage of intestinal neurons by various enteric glial cell‐related mechanisms. Some cytokines in the ENS involved in the neuroplasticity of enteric glial cells have been identified. Acetylcholine (ACh), the main excitatory neurotransmitter in the ENS, activates glial cells and is involved in the physiological regulation of gut processes.^67^ Myenteric enteric glia express muscarinic receptors (M3 and M5), and muscarine primarily activates M3R to promote intracellular Ca^2+^ signalling.^67^ Both glial cholinergic and purinergic receptors function via Ca^2+^‐dependent intracellular pathways. Moreover, responses in neuronal varicosities are triggered by the stimulation of neurokinin‐2 receptors (NK2Rs) and transmitted to enteric glia and neurons.^75^ The inhibition of NK2R signalling improves colitis, reactive gliosis, and neuroinflammation and prevents increased neuronal contractions.^75^

Growing evidence supports the interplay between glial cells in neuroplasticity and inflammation. Cytokines and chemokines are produced by reactive enteric glia; however, their functions have not been clearly established.^10^ PGE2 activation may enhance inflammation and is associated with tumorigenesis and the risk of intestinal cancer in CD.^76^ Bradykinin (BK), a potent algesic substance, is created from the plasma precursor kininogen in response to tissue damage, anoxia, or inflammation.^77^ The bradykinin B1 receptor (BKB1R) is largely expressed under pathological conditions and is frequently increased after tissue injury or exposure to bacterial endotoxins or cytokines.^77^ Furthermore, the classical inflammatory cytokines IL‐1β can affect neuronal excitability.^78^ IL‐1β up‐regulates BKB1R expression in glial cells, and the activation of BKB1R by BK increases Ca^2+^ production. As a result, the excitation of Ca^2+^ increases prostaglandin E2 (PGE2) release by the glia.^79^, ^80^

**FIGURE 2:**
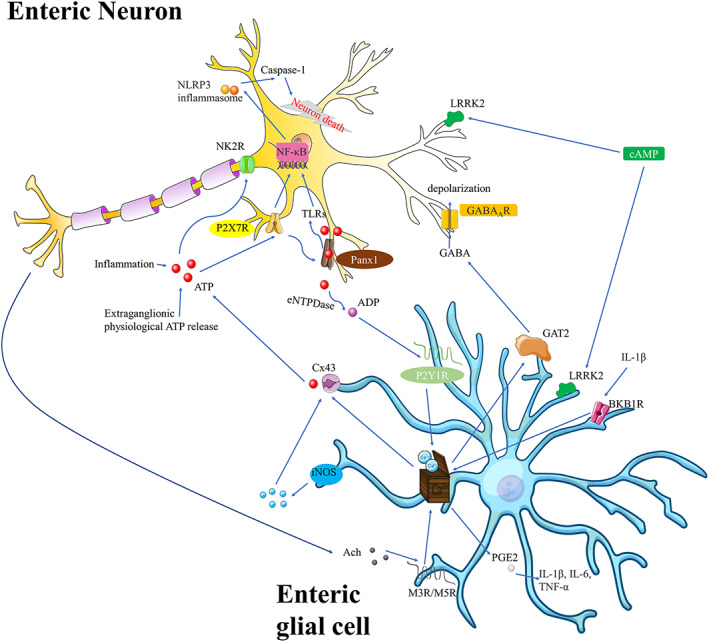
The neuroplasticity and neuromodulatory roles of enteric glial cells. During inflammation, ATP from NO produced by enteric glial cells can be transported to the P2X7R‐Panx1 pathway in enteric neurons and induce NF‐κB. The NLRP3‐inflammasome is activated, thereby promoting neuron death. Furthermore, ATP hydrolyzation stimulates Ca^2+^ responses in enteric glial cells, and Ca^2+^ triggers the release of gliotransmitters, including ATP and GABA. ACh‐induced glial activation and IL‐1β‐induced BKB1R activation also regulate Ca^2+^ signalling. Increased Ca^2+^ levels can release PGE2 and several pro‐inflammatory factors. Moreover, cAMP can affect LRRK2 levels in enteric neurons and glial cells.

Recent studies have provided new insight into the relationship between enteric glial cells and intestinal neurons. Leucine‐rich repeat kinase 2 (*LRRK2*), associated with Parkinson's disease,^81^ is a susceptibility gene for CD.^82^ LRRK2 levels are elevated in patients with CD, and furthermore, IFNγ can induce LRRK2.^83^ LRRK2 is upregulated in neurons and downregulated in enteric glial cells^84^; however, few studies have evaluated the specific mechanisms of LRRK2 in enteric glial cells. It is possible that LRRK2 produced by enteric glial cells regulates intestinal alpha‐synuclein aggregation and inflammation in CD.^85^, ^86^ Moreover, the cAMP pathway controls LRRK2 expression and phosphorylation in enteric glial cells^84^; however, its interactions with immune cells and specific regulatory mechanisms still need further elucidation. The differences in LRRK2 expression between neurons and enteric glial cells are remarkable and may be related to extracellular signal‐regulated kinases (ERK), since cAMP activates ERK in neurons but inactivates ERK in astrocytes.^87^ Therefore, LRRK2 may contribute to the pathophysiology of CD and also serve as a novel glial target for treatment. Enteric glial cells integrate information from neurons, immune cells, glial cells, and other cells in the gut environment via extensive networks using Ca^2+^ signals.^62^ Furthermore, enteric glial cells do not produce action potentials like neurons but can support neurons to modulate neural activity. The signalling pathways by which enteric glial cells participate in neuromodulation are illustrated in Figure 2.

## 4 ENTERIC GLIAL CELLS PROMOTE BARRIER INTEGRITY AND TISSUE REPAIR IN CD

Enteric glial cell activity is required for mucosal barrier integrity in vivo.^88^ Enteric glial cells influence the transcriptome and phenotype of epithelial cells to favour enhanced cell attachment and differentiation.^89^ Furthermore, they secrete crucial substances for gut barrier function. Notably, glial‐derived neurotrophic factor (GDNF),^90^ *S*‐nitrosoglutathione (GSNO),^88^ and 15‐hydroxyeicosatetraenoic acid (15‐HETE)^91^ levels are changed in CD.

GDNF regulates epithelial tight junctions in the intestinal epithelial barrier,^92^, ^93^ guards against bacterial infection of intestinal epithelial cells,^42^ and exerts anti‐inflammatory effects.^94^ The inflamed colons of patients with CD present significantly higher GDNF levels compared with noninflamed colons, which lack GDNF expression.^73^, ^95^, ^96^ However, lower glial fibrillary acidic protein (GFAP) and GDNF levels in CD compared with ulcerative colitis and infectious colitis indicate a weaker enteric glial cell network in this condition.^73^ One theory is that GDNF expression changes over the course of IBD and may depend on the level of inflammation.^97^ Meir et al.^98^ have indicated that enteric glial cells are a substantial source of GDNF. Enteric glial cells release GDNF at physiologically relevant doses, as reported in murine or human intestines both in vivo and in vitro.^98^ These dosages effectively promote barrier maturation and prevent damage to the intestinal epithelial barrier brought on by inflammation.^98^ Zhang et al.^94^ have found that GDNF can directly contribute to restoring epithelial barrier integrity in vivo by decreasing enhanced epithelial permeability and suppressing mucosal inflammatory response. Particularly, GDNF plays a significant role in mediating the interaction between mucosal epithelial cells and enteric glial cells.^94^ Under normal conditions, RET signalling is necessary to establish Peyer's patch and for intestinal stimulation by enteric neurons during the early stages of gastrointestinal tract development.^99^ Furthermore, RET in enteric neurons is exclusively activated by neurotrophic factors, including GDNF.^99^ RET activates IL‐22, linking intestinal epithelial defence with RET‐dependent ILC3 activation.^22^ IL‐22 enhances epithelial cell proliferation, survival, repair, and homeostasis in the intestine.^100^ It is believed that GDNF may play a protective role in chronic intestinal diseases, which suggests an involvement in CD. Additionally, it decreases epithelial permeability due to its anti‐apoptotic characteristics.^96^ Zonula occludens‐1 (ZO‐1) expression in colonic epithelial cells is upregulated by GDNF.^94^ GDNF‐mediated mucosal protection against apoptosis requires the activation of the mitogen‐activated protein kinase (MAPK) and phosphatidylinositol 3‐kinase/Akt (protein kinase B) pathways.^96^ CD is characterized by impaired intestinal epithelial barrier function with decreased desmosomal junctional protein desmoglein 2 (DSG2) via the RET‐p38 MAPK‐dependent phosphorylation of cytokeratins.^101^

It is worth emphasizing that enteric glial cells are not the only source of GDNF. GDNF is also produced and secreted by intestinal smooth muscle cells,^102^ neurons,^103^ skeletal muscle cells,^104^ and enterocytes,^92^ suggesting that these cells may be connected via an autocrine or paracrine signalling loop. Therefore, further studies are needed to determine which changes specifically originate from enteric glial cells. Furthermore, more in vivo evidence is required on the role of enteric glial cell specificity in CD.

GSNO, which is also produced by enteric glia, strengthens gut barrier integrity. After enteric glial cell disruption in the intestines of patients with CD, GSNO can maintain intestinal mucosal barrier function and protect against inflammation.^88^ In rats, GSNO reduced intestinal inflammation and epithelial barrier damage and protected against LPS‐induced inflammation, potentially by inhibiting the NF‐κB pathway.^105^ Increasing perijunctional F‐actin synthesis and the interaction between tight‐junction‐associated proteins, like ZO‐1 and occludin, and the actin cytoskeleton influence the GSNO‐mediated control of mucosal barrier function.^88^, ^106^ *Shigella flexneri*‐induced barrier lesions and inflammatory responses were dramatically decreased by enteric glial cells.^42^ Moreover, GSNO‐mediated effects were reproduced by the enteric glial cell, and the enteric glial cell‐protective effects were diminished by the pharmacological suppression of the pathways involved in GSNO synthesis.^42^

**FIGURE 3:**
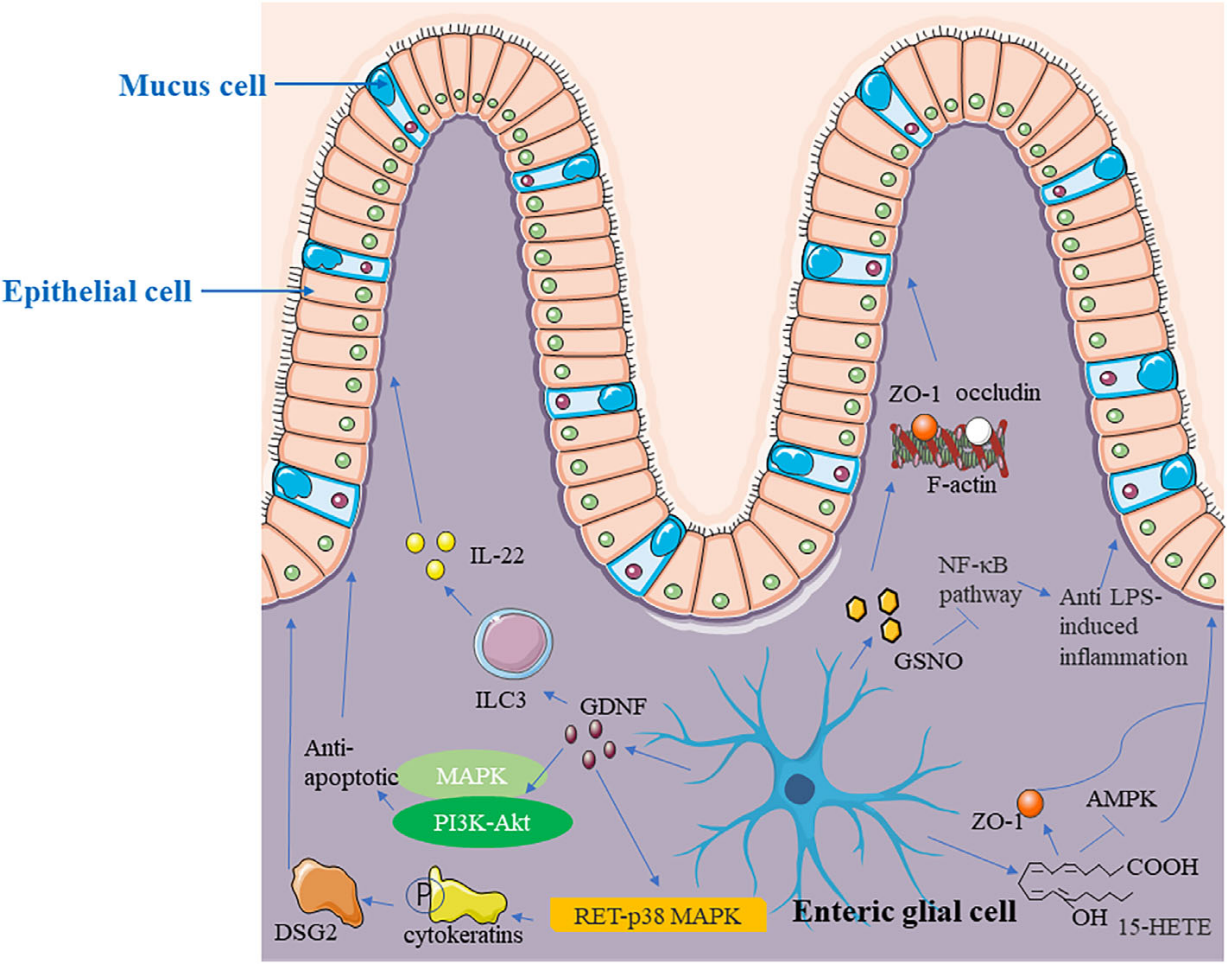
Enteric glial cells promote barrier integrity and tissue repair. First, enteric glial cells release GDNF, which activates ILC3, resulting in IL‐22 production. GDNF can also activate MAPK and PI3K‐Akt pathways to exert anti‐apoptotic effects in the epithelial barrier. GDNF upregulates DSG2 via the RET‐p38 MAPK‐dependent phosphorylation of cytokeratins. Moreover, enteric glial cells produce GSNO to assemble ZO‐1 and occludin with the actin cytoskeleton. GSNO also inhibits the NF‐κB pathway to protect against LPS‐induced inflammation. Enteric glial cells promote 15‐HETE production to increase ZO‐1 levels and inhibit AMPK expression.

In addition, a polyunsaturated fatty acid metabolite produced by enteric glial cells, 15‐HETE, promotes intestinal epithelial barrier expansion and resistance and reduces intestinal epithelial barrier permeability. In patients with CD, enteric glial cells produce less 15‐HETE. Further, ZO‐1 expression is increased, and adenosine monophosphate‐activated protein kinase (AMPK) is inhibited by 15‐HETE, which is necessary to control epithelial resistance and permeability.^91^ Enteric glial cells may have tissue‐repair and barrier‐protective roles during CD development. The relevant mechanisms are summarized in Figure 3, which focuses on the roles of glial mediators, such as GDNF, GSNO, and 15‐HETE, released by enteric glial cells in the intestinal mucosal barrier. Future studies should explore the comprehensive mechanisms of enteric glia in promoting barrier integrity.

Although many researchers have found that enteric glial cells have important roles in the intestinal mucosal barrier, contrary opinions are also emerging. In transgenic mouse models, enteric glial cells cannot acutely affect barrier function.^107^ Additionally, both germ‐free mice and mice administered antibiotics lack mucosal glial cells.^108^ In animal models of colitis, NO released by enteric glia caused damage to epithelial barrier function.^31^ Mice are able to tolerate a significant loss of enteric glia cells, which leaves room for various interpretations regarding the extent to which enteric glia affect the epithelial barrier. In brief, it appears that enteric glia cells do not play a crucial role in regulating epithelial barrier function, or at least, they are not indispensable.

## 5 PROSPECTIVE CD THERAPIES TARGETING ENTERIC GLIAL CELLS AND MECHANISMS

Enteric glia serve as a possible therapeutic target for a variety of gastrointestinal illnesses owing to their neuromodulatory and immunomodulatory abilities; however, the development of cell‐specific therapies is limited. Notably, there are no treatments or medications that have been developed to specifically influence glial functions; however, glial mechanisms can be affected by some CD therapies. This suggests that some CD therapies might exert effects on glial cells.^10^ The CD therapies that possibly target enteric glial cells are listed in Table 1.

**TABLE 1:** Candidate CD therapies targeting enteric glial cells and their functions.

Glial mechanism	Target	Available drugs	Functions in glial cells
Neuron–glia interaction	P2X7R/Panx1	P2X7R antagonists: AZD9056, GSK1482160; Panx1 antagonist: spironolactone	Prevents neuronal apoptosis; protects against colonic motor impairment induced by inflammation; reduces neuroinflammation and visceral hypersensitivity (pain)
Glial–immune interaction	TLR4	Low‐dose naltrexone	Anti‐inflammatory effects targeting TNF‐α and IL‐6; analgesic effect
Glial–immune interaction	S100B/RAGE/NO	RAGE inhibitor: *Brucea javanica* oil emulsion (BJOE); S100B inhibitor: pentamidine	Restores intestinal epithelial barrier function and lowers bacterial translocation; prevents inflammatory reactions (e.g., by reducing NF‐κB and IL‐6); prevents proliferation, autophagy, and apoptosis; reduces reactive gliosis; relieves visceral pain
Neuron–glia interaction; glial–immune interaction; promoting epithelial barrier integrity	GABA	Glutamine	Ammonia detoxification; reduces bacterial translocation; lessens inflammation and disease activity; maintains the integrity of the intestinal mucosa
Glial–immune interaction; promoting epithelial barrier integrity	NF‐κB pathway (TNF‐α, IL‐12/23, IκBα)	Anti‐TNF antibodies: infliximab, adalimumab, certolizumab; Anti‐IL‐12/23 antibodies: ustekinumab; IκBα activator: corticosteroids	Maintains intestinal immunological homeostasis and decreases chronic inflammatory processes in the intestines; intestinal epithelial barrier repair
Promoting epithelial barrier integrity	GDNF	None	Protects the epithelial barrier and decreases pro‐inflammatory cytokine release

The P2X7R‐Pannexin‐1 signalling pathway has been gaining increased attention. Evidence indicates that purinergic signalling contributes to the pathophysiology of intestinal inflammation, and P2X7R is a promising new therapeutic target for CD.^109^ AZD9056, a P2X7R antagonist, reduced symptoms in patients with moderate‐to‐severe CD in a randomized placebo‐controlled, multicenter, double‐blind, phase IIa study.^110^ Further, P2X7 antagonism may be beneficial for the treatment of persistent abdominal pain, despite the lack of change in inflammatory indicators.^110^ Other potential drugs, like GSK1482160, are being evaluated in clinical trials.^111^ Of note, some researchers have found that P2X7R inactivation can not only decrease inflammation in IBD but also increase the incidence of colitis‐associated cancer in a mouse model.^112^ Therefore, it is advisable to proceed cautiously while using P2X7R antagonists to treat CD.^113^, ^114^

Inflammatory cytokine‐induced cell destruction, loss of the tight junction barrier, and increased permeability were reduced by a Panx1 channel blocker, suppressing colonic inflammation.^115^ The Panx1 antagonist spironolactone, used to treat hypertension,^116^ is also a candidate for CD treatment. In rats with CD, the traditional Chinese therapy herb‐partitioned moxibustion inhibited the P2X7R‐Panx1 signalling pathway to decrease excess NLRP3 inflammasome activation, in turn reducing the release of the inflammatory cytokines IL‐1β and IL‐18.^117^ The Panx1 antagonist is also a pain reliever that can decrease oxaliplatin‐induced neuropathic pain without reducing its efficacy in eliminating HT‐29 colon cancers cells.^118^ It is proposed that blocking the Panx1 channel may provide a new target in chronic pain control^119^ that may relieve symptoms in CD.

TLR4, which is present in glia, is a non‐opioid receptor antagonized by naltrexone.^120^ Low‐dose naltrexone reduces objective indicators of inflammation (i.e., TNF‐α and IL‐6^121^), disease severity, and self‐reported discomfort.^122^ Moreover, naltrexone decreases the Crohn's Disease Activity Index (CDAI), promotes mucosal healing,^123^, ^124^ and might effectively reduce inflammation by restoring normal enteric glial cell function.^62^

Similarly, targeting S100B/RAGE and NO generation in enteric glial cells is a potential therapeutic approach for CD. Blocking the RAGE system can prevent inflammatory reactions, proliferation, autophagy, and apoptosis, suggesting that it is a useful therapeutic strategy for CD.^62^ In mice with colitis, blocking intestinal glial activity by reducing NO synthesis restored intestinal epithelial barrier function and lowered bacterial translocation.^31^ *Brucea javanica* oil emulsion (BJOE), a traditional Chinese medicine, can inhibit RAGE expression and TLR4/NF‐κB‐mediated inflammatory responses, suggesting that it is a novel therapeutic option for CD treatment.^125^ Pentamidine, an S100B inhibitor, can reverse the effect of 5‐fluorouracil on enteric glial cell activation, neuronal loss, and intestinal inflammation.^126^ Because intestinal inflammation involves S100B overexpression in enteric glial cells and may affect pain signal pathways by several cytokines and neuromodulators,^51^ enteric glia may serve as a potential target for ameliorating pain in CD.^127^

Glutamine may improve CD by reducing intestinal inflammation. Furthermore, glutamine also reduces bacterial translocation and weakens inflammation and disease activity in experimental animal models of CD.^128^ Enteric glial cells express glutamine synthetase,^5^ which regulates ammonia detoxification and alters neuromuscular transmission through GABAergic signalling.^72^ Glutamine is essential for maintaining the integrity of the intestinal mucosa and increasing IL‐10 synthesis, while simultaneously decreasing the production of the pro‐inflammatory cytokines IL‐6 and IL‐8.^129^ However, the efficacy and safety of glutamine for CD treatment have not been clearly established.^130^, ^131^

Many drugs for the treatment of CD involve the NF‐κB signalling pathway, which is expressed in enteric glial cells. Numerous pro‐inflammatory genes that encode cytokines, chemokines, and adhesion molecules crucial for inflammation and immunological responses are stimulated by NF‐κB.^132^ The NF‐κB signalling pathway regulates the expression of TNF‐α, IL‐1, IL‐6, IL‐12, IL‐23, and IκBa.^133^ Anti‐TNF antibodies, including infliximab, adalimumab, and certolizumab,^134^, ^135^, ^136^ are a first‐line treatment for CD and several other autoimmune illnesses.^137^ Other biologic medications include IL‐12/23 antibodies, e.g., ustekinumab,^138^ and corticosteroids, which exert immunosuppressive effects by increasing IκBα, a crucial component of the NF‐κB pathway.^133^ In addition to immunomodulatory effects, anti‐TNF therapy could increase IL‐22 production in patients with CD and consequently promote intestinal epithelial barrier restoration.^139^ Notably, these anti‐inflammatory medications do not directly act on glia, and the specific relationship between these medications and enteric glial cells remains to be further studied.^137^

GDNF promotes epithelial barrier integrity. Under pathological circumstances, the loss of epithelial barrier integrity permits the translocation of luminal antigens into the mucosa, triggering the release of pro‐inflammatory cytokines. These cytokines break down the mucosal barrier and increase mucosal permeability, which could enable the translocation of bacteria.^94^ Accordingly, interrupting this cycle is a potential strategy for CD treatment. In a murine model of colitis induced by dextran sulphate sodium, GDNF reduced epithelial permeability as well as TNF‐α and IL‐1β production, inhibited the intestinal inflammatory response, and weakened disease severity. At the early stages of CD, GDNF may promote mucosal repair, and elevated levels of circulating GDNF may be linked to relapse.^97^ In conclusion, GDNF mediates the interaction between mucosal epithelial cells and enteric glial cells and may be a therapeutic target in CD treatment.

Enteric glial cells are a possible therapeutic target owing to their crucial involvement in maintaining neuron function, immune homeostasis, and barrier integrity. However, enteric glial cells also have multiple roles in tumorigenesis and can active colon cancer stem cell‐driven tumorigenesis.^140^ Moreover, enteric glial cells serve as the initial entrance site to the CNS in many infections, such as John Cunningham virus infection^141^ and prion disease.^142^ Therefore, the specificity of glial cell signalling in the digestive tract and whether targeting glial gains outweighs the drawbacks remain open questions.

In this review, we list several therapies that involve one or more targets on the glial pathway. As noted above, these targets are not only present in glial cells, and further research should specifically target glial cells. Thus, investigating the therapeutic potential of enteric glial cells is expected to drive further fundamental and translational studies.

## 6 CONCLUSION

The pathogenesis of CD is complex and involves many enteric cells. This review emphasizes the role of enteric glial cells in CD. Links between enteric glial cells and IBD have been reported;^10^, ^62^, ^143^ however, despite this, little is known about the crosstalk between cells and the underlying pathways through which enteric glial cells contribute to CD, emphasizing the importance of a detailed inventory of relevant signalling pathways.

Enteric glial cells are involved in various gastrointestinal diseases, such as IBD, postoperative ileus, irritable bowel syndrome, and motility disorders.^62^ Enteric glial cells regulate gut homeostasis by influencing mucosal permeability, neuronal activity, immunological activity, motility, endocrine secretion, absorption, and vascular tone.^62^ In this review, we emphasize three key roles of enteric glial cells in CD: neuroplasticity, immune homeostasis, and barrier integrity. These three functions are not mutually exclusive. For example, GDNF^144^ is not only a glial‐derived barrier‐enhancing factor^92^ but also activates proinflammatory cytokines and acts as an intermediate between glial cells and lymphocytes.^22^

Increasing evidence indicates that enteric glial cells are a key component in the intestinal immune response against invading microorganisms. Enteric glial cells release and react to a variety of cytokines and chemokines, express TLR, RAGE, and MHC II, interact with macrophages, NK cells, and other immune cells, and are in close proximity to lymphocytes and other inflammatory effectors. A recent study has shown that the gut microbiota is essential for sustaining the formation of new enteric glial cells in the gut and demonstrated the strong link between enteric glial cells, bacteria, and the microbiome.^108^ Future studies should focus on the role of enteric glial cells in the intestinal microenvironment and ENS as well as their immune capacity. Advanced genetic and imaging tools provide a basis for determining the exact roles of enteric glial cells and have improved our understanding of the significance of the glial Ca^2+^ response in the pathogenesis of CD. However, interactions between enteric glial cells, neurons, and non‐neuronal cells are complex and not fully understood. The molecular mechanisms by which enteric glial cells affect neurons or immune cells and thereby regulate gastrointestinal functions require further investigation.

Disruption of intestinal barrier integrity results in inflammation and tissue damage. Here, we reviewed several factors released by enteric glial cells that promote intestinal integrity in detail, including GDNF, GSNO, and 15‐HETE. Several other molecules may also be involved in CD formation. For example, transforming growth factor‐β1 (TGF‐β1) and 15‐deoxy‐Δ12,14‐prostaglandin J2 (15dPGJ2), which are produced by enteric glial cells and involved in mucosal barrier function,^89^ may be involved in CD. A recent study has demonstrated that glial cells express TLR2, which is essential for recognizing microbial structures.^145^ TLR2 also has cytoprotective effects on intestinal epithelial cells by increasing GDNF expression.^146^ CD involves dysbiosis,^1^ and alterations in the microbiota in CD are a major focus of studies. Future studies are expected to focus on how enteric glial cells act on the intestinal barrier.

Enteric glial cells are emerging as possible therapeutic targets. Enteric glia pathways provide direction for targeted therapies, including the P2X7R‐Panx1, S100B‐RAGE‐TLR‐NO, Ca2 + ‐GABA, NF‐κB, and GDNF pathways. Drugs that relieve ulcerative colitis could also serve as treatments for CD, as these drugs usually relieve inflammation. For example, palmitoylethanolamide can reduce TLR4 and S100B expression, inhibit NF‐κB via the p38/p‐ERK/pJNK pathway, and reduce the production of proinflammatory factors.^29^ Enteric glia pathways can contribute to various intestinal diseases, including CD and ulcerative colitis. Nonetheless, targeted drugs may show different efficacy in these two diseases. Therefore, more clinical studies are needed to explore the sensitivity to specific targeted therapies.

Recently, increasing single‐cell resolution studies on IBD have been published, providing new perspectives in research on enteric glia cells. In an RNA sequencing profile comparing colonic mesenchyme between healthy and ulcerative colitis mice, glial cells were typically clustered by the expression of their markers S100B and GFAP, and possible additional markers include Hapln1 and POSTN.^147^ With the aid of these markers, the differences in glial cells gene expression between healthy and inflamed bowel samples were discussed. Another study that utilized samples from the human colon employed S100B, CD68, XCR1, and CLEC9A as markers^148^ and demonstrated that the proportion of glial cells in inflamed colon samples was lower than in the healthy samples. Among putative IBD risk genes, glial cells show high expression of *ZNF831* (which participates in the HNF4A pathway), *C7orf72* (which initiates the IRf5 pathway) in the inflamed colon, as well as elevated expressions of *PRKCB* and *PLCG2* (which participate in the IRF8 pathway together), *BACH2* (which participates in the TAB2 pathway to recruit TAF8), *PTPRC* (which participates in the IRF6 pathway), and *IKZF1* (which participates in the IRF5 pathway) in both healthy and inflamed colon samples.^148^ This study has also shown that glial cells exhibit low anti‐TNF therapy sensitivity and resistance (low response and a minimal relationship with resistance) compared with other components of the colon mesenchyme. Furthermore, no downregulated risk genes were detected. The *LTB* and *KYNU* genes are among others that are considerably differentially expressed in glial cells.^148^ To elucidate the phenotypic differences in glial cells within inflamed colons, a study employed bacterial LPS and IFN‐γ treatment to induce an inflamed state in human enteric glial cells,^149^ and the most prominent change involved the disruption of Ca^2+^ responses and purinergic signalling. This result indicated that the inflammatory response in human enteric glial cells is closely linked to alterations in the purinergic signalling pathway. Moreover, it is induced by a change in the expression levels of calcium channels, like CHRNA7, and the purinergic gene *Adora2a*, accompanied by changes in the expression of transcriptional factors like RELA and RELB.^149^ This may indicate that the calcium response and purinergic pathway may be targets for glial cell abnormalities in IBD.^149^ These sequencing studies provide several novel possibilities for the future targeting of enteric glial cells in IBD, especially CD.

New research on the connection between enteric glial cells and the pathogenesis of CD has considerable theoretical and clinical implications. More slide‐seq, single‐cell RNA sequencing, and RNAscope studies are needed to elucidate specific enteric glia subtypes and assess various pathway changes in different gastrointestinal diseases. Comprehensive documentation on the poorly understood area of enteric glial immune capacity and glial‐specific immune‐modifying signalling pathways is limited. Further, the specificity of glial signalling in CD remains poorly understood and requires more in vivo evidence. Future research should focus on CD susceptibility genes, enteric glia characteristics, and targeting enteric glial cells in CD.

## AUTHOR CONTRIBUTIONS

Xinyi Mao collected the paper and data, made conclusion analysis and drafted the manuscript; Jun Shen presented the idea of this paper, supported the funding, made conclusion analysis and drafted and revised the manuscript.

## CONFLICT OF INTEREST STATEMENT

The authors of this manuscript declare no conflict of interest.

## Data Availability

Data sharing not applicable to this review as no datasets were generated or analysed during the current study.
